# Dental pain report in children and genetic polymorphism (rs4818) in Catechol-O-Methyltransferase (*COMT*) gene: a cross- sectional study

**DOI:** 10.1590/1678-7757-2023-0229

**Published:** 2024-01-05

**Authors:** Bruna Leticia Vessoni Menoncin, Aluhê Lopes Fatturi, Rafaela Scariot, José Vitor Nogara Borges Menezes, João Armando Brancher, Juliana Feltrin-Souza

**Affiliations:** 1 Universidade Federal do Paraná Departmento de Estomatologia Curitiba PR Brasil Universidade Federal do Paraná, Departmento de Estomatologia, Curitiba, PR, Brasil.; 2 Universidade Positivo Curitiba Brasil Universidade Positivo, Programa de Pós-Graduação em Odontologia, Curitiba, Brasil.

**Keywords:** Genetic polymorphism, Pain perception, Toothache, Child, Pediatric dentistry

## Abstract

**Aim:**

Polymorphisms in the COMT gene can alter enzymatic functions, raising levels of endogenous catecholamines, which stimulates beta-adrenergic receptors related to pain. This study aimed to evaluate whether a polymorphism in the *COMT* gene (rs4818) is associated with dental pain in children.

**Methodology:**

A cross-sectional study was conducted with a representative sample of 731 pairs of children and parents randomly selected from a population-based sample of eight-year-old children. Reports of dental pain was evaluated using a question directed at the parents and self-reported pain using the Faces Pain Scale – Revised. Dental caries experience was determined using the Decayed, Missing, and Filled Teeth (DMFT) index. For genetic analysis, DNA was obtained from oral mucosa epithelial cells of 352 children randomly selected from the initial sample.

**Results:**

Children with the CC genotype had higher odds of reporting moderate to intense pain than those with the GG genotype (OR=3.60; 95% CI=0.80–16.20; p=0.03). These same children had greater odds of parental reports of pain (OR=1.93; 95% CI=0.91-4.08; p=0.02). Moreover, lower schooling of parents/guardians and caries experience in the primary dentition were significantly associated with greater odds of a parental report of dental pain (OR=2.06; 95% CI=1.47–2.91; p<0.001; OR=6.26; 95% CI=4.46–8.78; p<0.001).

**Conclusions:**

The rs4818 polymorphism of the *COMT* gene is associated with dental pain. Children with the C allele are more likely to report higher levels of pain. Clinical Relevance: Even though the experience of pain is subjective and multifactorial, this study raises the hypothesis that there is a genetic predisposition to dental pain that should be considered in clinical practice.

## Introduction

According to the International Association for the Study of Pain (IASP), pain is defined as an unpleasant sensory and emotional experience associated with actual or potential tissue damage or described in such terms.^1^ The evaluation of the pain experience is entirely subjective^2,3^ and characterized by individual variability, as it depends on the emotional or psychological status.^4^ In fact, the biological basis for pain perception involves complex interactions and includes individual genetic susceptibility.^5^

Regarding pain perceived in the face and oral cavity, pain caused by dental caries is the most common^6–8^ and is the main reason for children to visit the dentist.^9^ In addition, the pain stemming from this condition can exert a substantial negative impact, especially in children.^10^ Dental pain is a typical example of protective pain, indicating acute or chronic inflammation that persists indefinitely until the aggressor agent is removed. Nonetheless, until this happens, the injury and the inflammatory process trigger a cascade of molecular, immunological, and hormonal events that culminate in the release of neurotransmitters^11^ which trigger the modulation of the inflammatory response.^12,13^

The whole process of dental caries leading to dental pain is usually of multifactorial etiology and rarely due to only one phenomenon. In fact, dental caries and dental pain are influenced by biological, behavioral, social, and environmental factors^14^ and are strongly associated with individual genetic background.^15^ In this sense, polymorphisms located in *pain genes* are candidates to be studied in individuals who report pain sensitivity. One of these pain genes is Catechol-O-Methyltransferase (*COMT*) gene, located on chromosome 22, and consists of six exons and two promoter regions^16^ that encode COMT proteins with a highly specialized enzymatic function that performs the reuptake of catecholamines released into the synaptic gap, modulating the signals transmitted by these neurotransmitters and contributing to analgesia.^17^

In humans, many polymorphisms in the *COMT* gene have been associated with emotional disorders, such as stress,^18^ pain conditions,^19,20^ including chronic pain,^21^ and temporomandibular disorders.^22^ One of these polymorphisms of particular interest is rs4818, located in exon 4, that promotes a C/G substitution in a specific region of the *COMT* gene that codified the expression of the two enzymes involved in analgesia.^23^ To the best of our knowledge, there has been no investigation into the association between dental pain and this polymorphism. Therefore, in this study, we tested the hypothesis that the rs4818 polymorphism is associated with the perception of dental pain in a Brazilian children population.

## Methodology

### Ethical considerations

This study received approval from the Human Research Ethics Committee of the Federal University of Paraná (certificate number: 1.1613.829) and authorization from the Municipal Secretary of Education. Parents/guardians were informed about the objectives and procedures of the study and authorized their children’s participation by signing an informed consent form. This study was conducted in accordance with the precepts stipulated in the Declaration of Helsinki and was reported using the STREGA guidelines.

### Participants

A population-based cross-sectional study was conducted with a representative sample of 731 pairs of children and their parents, randomly selected from a representative population-based sample of eight-year-old children.^24^ Data collection and participant selection took place from November 2016 to September 2017 at public schools in the city of Curitiba, state of Paraná, Brazil.

The sample size calculation was previously described in the two manuscripts published by our research group.^24,25^ It was based on a finite population, the outcome proportion was set at 50%, an accuracy of 5% was used, and a design effect factor of 1.8 was established for cluster/sampling. A boundary of 1.96 (20%) for the rejection area was added to compensate for occasional losses, resulting in a final sample of 690 to 865 children. Eight-year-old students with at least one first permanent molar erupted were included in this study. Those who wore an orthodontic appliance at the time of the clinical examination and those with any syndrome that impeded the examination were excluded.

### Reports of pain of a dental origin

The parents’/guardians’ reports of pain were recorded based on their answer to the following question: “Has your child ever had a toothache?”

Self-reported pain was evaluated using the Faces Pain Scale – Revised (FPS-R),^27^ which is a scale with good psychometric properties for the four- to 17-year-old age group with acute pain. The scale is composed of six faces that represent increasing intensities of pain. The scale is administered with standardized statements available online in several languages at the IASP electronic address. The Brazilian Portuguese version was validated by Tomlinson in 2010.^28^ The linear score ranges from zero to 10. The following statements were given to the children: “These faces show how much something can hurt. This face (pointing to the image on the far left) shows no pain. The other faces show more and more pain (pointing to each figure from left to right) until this one (pointing to the last image). It shows a lot of pain. Point to the face that shows how much you hurt right now.” The interviewer marks the chosen face, which is scored 0, 2, 4, 6, 8, or 10 (from left to right), with “0” meaning no pain and “10” meaning maximum pain.^28^

### Collection of clinical data

For the clinical examination, dental caries experience was evaluated using the Decayed, Missing, and Filled Teeth (DMFT) index for primary and permanent teeth, recommended by the World Health Organization.^29^ Four examiners had previously undergone training and calibration exercises in a clinical setting with 20 children, achieving a good level of agreement (≥ 0.75).^26^ Those children were not included in the main study.

Data on the schooling of the parents/guardians were collected using a structured question about the number of years of formal schooling education, as a possible interaction variable between the report of pain and other variables. Prior to the data collection process, a pilot study was conducted at a school in the city with 80 children of the same age and with similar conditions. The results revealed no need to alter the questionnaire.

### Genetic polymorphisms

**Figure 1:**
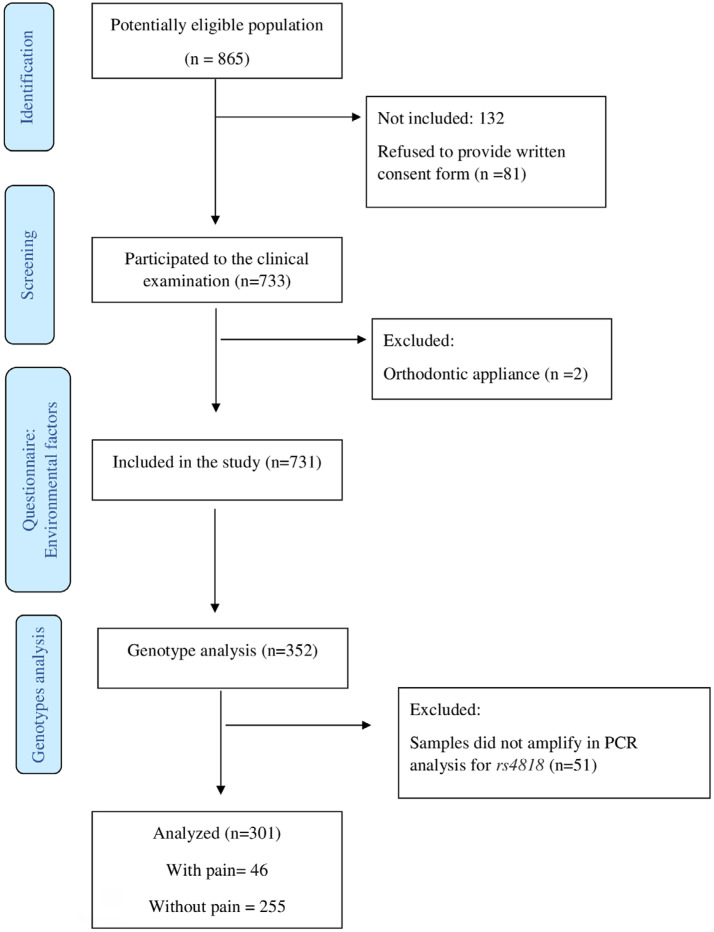
Flow diagram of study. Individuals with scores 6, 8 and 10 on the FPS-R were considered to be with pain. Case-group included children with at least one second primary molar affected by Hypomiralised Second Primary Molars. Control group included children without HSPM

For the genetic analysis, 352 children were randomly selected from the initial sample that was enrolled in the previous case-control design (Figure 1).^24^ In the case group, all children presenting with molar-incisor-hypomineralization (MIH) according to the European Academy of Paediatric Dentistry (EAPD) criteria were included. For the control group, children without enamel defects were randomly selected and matched by age, ethnicity, and gender in a ratio of 3:1 (case:control). The same sample was analyzed regarding dental pain and genetic polymorphisms. DNA was obtained from oral mucosa epithelial cells using a mouthwash consisting of 5 ml of a 3% glucose solution, following a previously published protocol.^31^ The polymorphism in the *COMT* gene (rs4818; flanking sequence in the GAGGCT[C/G]ATCACCT context; global minor allele frequency = 0.37; located at Chr.22:19963684) was selected for analysis due to the association with pain (data from www.snpedia.com and https://www.thermofisher.comwebsites).

The allelic discrimination analysis and genotyping were performed using real-time polymerase chain reaction and the Taqman test (GeneAmp PCR System 9700, Thermo Fisher Scientific, Waltham, MA, USA). The chi-square test was used to calculate whether the polymorphism was in Hardy-Weinberg equilibrium.

### Statistical analysis

Statistical analysis was performed using Statistical Package for the Social Sciences 16.0 (SPSS, CHICAGO, IL, USA) and STATA 14.0 (StataCorp, College Station, TX, USA). The dependent variables were self-reported dental pain based on the results of the FPS-R and categorized as ”none or little” (scores of 0, 2, and 4) and “moderate to intense” (scores of 6, 8, and 10)^31^ and parental reports of dental pain based on the answer to the question “has your child ever had a toothache?” (no = 0; yes = 1). The independent variables were schooling of the parents/guardians, which was dichotomized as < eight years or > eight years of study (corresponding to primary school in Brazil), dental caries, which was dichotomized as present (untreated dental caries lesion (component D) ≥ 1) or absent (untreated dental caries lesion (component D = 0), and genotype, which was categorized as additive, dominant allele, or recessive allele. Odds ratios (OR) and 95% confidence intervals (CI) were calculated for the analysis of associations, considering a p-value ≤ 0.05 as indicative of statistical significance.

## Results

The response rate was 90.6% of the sample invited to participate in the study. The clinical examination was performed on 733 children, two of whom were excluded for not meeting the eligibility criteria. Thus, the final sample comprised 731 children, 374 (51.1%) boys. For the genetic analysis, of the eligible children (n=352), 255 were included in the control group, and 46 were included in the case group, so that the ratio was 5.5:1 (control/case). DNA samples of 51 children were excluded because they did not amplify in the polymerase chain reaction (PCR) analysis. Regarding genetic variation, the dominant allele frequency was constant in the population studied, i.e., the polymorphism is in Hardy-Weinberg equilibrium.

Table 1 displays the associations between self-reported pain and the characteristics of the population analyzed, including genetic polymorphism. A statistically significant association was found between self-reported pain and the polymorphism (*rs4818*). In the analysis of the genotype model, children with the CC genotype were 3.6 times more likely to report moderate to intense pain than those with the GG genotype (OR = 3.60; 95% CI: 0.80 to 16.20; p=0.03). The association with the CC or GC genotype in the dominant model remained statistically significant compared to the GG genotype (OR = 4.33; 95% CI: 1.01 to 18.58; p=0.04). No statistical difference was observed in the recessive model.

**Table 1:** Association between self-reported pain and sample characteristics (N=305; Curitiba, PR – Brazil, 2017)

CHARACTERISTICS		FPS-R		Total	OR (95%CI)	p
		Moderate to intense	No or low pain	N		
		n (%)	n (%)			
Schooling of the parents/guardians	≤ 8 years	39 (19.0)	166 (81.0)	205 (28.3)	1	0.08
	> 8 years	72 (13.9)	446 (86.1)	518 (71.7)	1.45 (0.94–2.23)	
Decayed	0	47 (13.2)	314 (86.8)	361 (49.4)	1	0.08
	≥ 1	65 (17.6)	305 (82.4)	370 (50.6)	1.45 (0.94–2.23)	
rs4818	GG	2 (4.5)	42 (95.5)	44 (14.6)	1	–
	GC	26 (19.4)	108 (80.6)	134 (44.5)	3.60 (0.80–16.20)	0.09
Additive model	CC	18 (14.6)	105 (85.4)	123 (40.9)	5.05 (1.14–22.24)	0.03*
rs4818	GG	2 (4.5)	42 (95.5)	44 (14.6)	1	0.04*
Dominant model	CC/GC	44 (17.1)	213 (82.9)	257(85.4)	4.33 (1.01–18.58)	
rs4818	CC	18 (14.6)	105 (85.4)	123(40.9)	1	0.79
Recessive model	GC/GG	28 (15.7)	150 (84.3)	178(59.1)	0.91 (0.48–1.74)	

Note: FPS-R (Faces Pain Scale – Revised); n (Total of individuals); OR (Odds ratio); CI (Confidence interval); p (p value);

* Highlighted values represent statistical significance (p≤0.05).

Table 2 displays the association between parental reports of dental pain and the independent variables. The association between the pain report and the *COMT* gene polymorphism (rs4818) was statistically significant. Children with the CC genotype were more likely to have a parental report of dental pain than those with the GG genotype (OR = 1.93; 95% CI: 0.91 to 4.08; p=0.02). Moreover, lower schooling of parents/guardians and caries experience in the primary dentition was significantly associated with a greater likelihood of a parental report of dental pain (OR = 2.06; 95% CI: 1.47 to 2.91; p<0.001; OR = 6.26; 95% CI: 4.46 to 8.78; p<0.001, respectively). Once again, no significant statistical difference was observed when the recessive model was used.

**Table 2:** Association between parental report of pain and sample characteristics (N=305; Curitiba, PR – Brazil, 2017)

CHARACTERISTICS		PARENTAL REPORT OF PAIN		Total	OR (95%CI)	p
		With pain n(%)	Without pain n(%)	N		
Schooling of the parents/guardians	≤ 8 years	105 (56.2)	82 (43.8)	187(27.6)	1	<0.001*
	> 8 years	187 (38.2)	302 (61.8)	489(72.4)	2.06 (1.47–2.91)	
Decayed	0	40 (14.5)	261 (85.5)	335(49.1)	1	<0.001*
	≥ 1	222 (64.0)	125 (36.9)	347(50.9)	6.25 (4.46–8.78)	
rs4818	GG	13 (31.0)	29 (69.0)	42(15.0)	1	–
Additive model	GC	63 (50.4)	62 (49.6)	125(44.6)	1.93 (0.91–4.08)	0.08
	CC	52 (46.0)	61 (54.0)	113(40.4)	2.27 (1.08–4.75)	0.02*
rs4818	GG	13 (31.0)	29 (69.0)	42(15.0)	1	0.03*
Dominant model	CC/GC	115 (48.3)	123 (51.7)	238(85.0)	2.10 (1.04–4.23)	
rs4818	CC	52(46.0)	61(54.0)	113(40.4)	1	0.93
Recessive model	GC/GG	76(45.5)	91(54.5)	167(59.6)	1.02 (0.63–1.64)	

Note: n (Total of individuals); OR (Odds ratio); CI (Confidence interval); p (p value);

* Highlighted values represent statistical significance (p≤0.05).

## Discussion

This cross-sectional study aimed to evaluate a probable association between the rs4818 polymorphism and the perception of pain of dental origin in a Brazilian children population. The main results provide evidence that the presence of the C allele in this polymorphism is associated with increased reports of dental pain perception in the population studied.

Since the genetic approach has been used to explain painful conditions, the *COMT* gene has emerged as a strong candidate for study, as it is considered one of the “pain genes.”^32^ Since this gene encodes two essential enzymes that act in the reuptake of neurotransmitters involved in pain perception, the first is called Soluble COMT (S-COMT), present in the cell cytoplasm; the second is called Membrane-bound COMT (MB-COMT), a protein bound to the plasma membrane.^33^ Polymorphisms in the *COMT* gene can change the three-dimensional conformation of these enzymes and decrease their activity,^34^ so the pain perception is different. This information explains the different degrees of pain intensity perceived by individuals, suggesting that individual genetic background may play an important role in pain perception.^35^

Among the dozens of polymorphisms in the *COMT* gene, three polymorphisms are the most widely studied: rs4633, rs4818, and rs4680, all situated in the region that codes the expression of the two enzymes (S-COMT and MB-COMT). Among these polymorphisms, rs4680 is the most documented, as an amino acid is substituting in the protein structure that generates enzymes with less thermostability, leading to a reduction in enzymatic activity and, consequently, a greater perception of pain.^34–38^ In this study, the chosen rs4818 polymorphism could have a significant clinical impact. It is located in the coding region denominated exon 4, which is very close to rs4680, leading us to assume that it could contribute to the structural change in the enzymes. In fact, these results showed that reported experiences of moderate to intense pain were much more frequent among individuals with the CC or CG genotype than among those with the GG genotype. Parental reports of pain experience were also more frequent among children with the CC or CG genotype. Thus, both self-reports and parental reports of dental pain were associated with the rs4818 polymorphism, and it seems that the C allele is a determinant factor in pain perception, meaning that pain perception is lower in individuals with the G allele. Clinically, this information suggests that some individuals may report more or less pain of dental origin due to the polymorphism.

This genetic polymorphism has been associated with the experience of pain, especially chronic pain, and has been discussed by other authors.^35–38^ Lee, et al.^35^ (2011) found that the presence of the G allele was associated with less pain sensitivity following the extraction of third molars. Smith, et al.^36^ (2011) also found an association between the *COMT* gene and both chronic pain and temporomandibular disorder, concluding that the G allele is related to lower activity of the *COMT* gene (rs4680), which increases the pain threshold, making it a protective factor. Similar results have also been reported for myofascial pain in adolescents. Those with the C allele were more predisposed to episodes of pain.^39^ Sagud, et al.^37^ (2018) found that the presence of the G allele of rs4818 is associated with more significant activity of the *COMT* gene, affecting the levels and function of dopamine in the frontal cortex region. As this activity is inversely associated with pain sensitivity, individuals with the GG genotype have high enzyme activity and therefore low pain sensitivity. In contrast, those with the CG genotype have intermediate pain sensitivity, and those with the CC haplotype have low enzyme activity and high pain sensitivity. This association was demonstrated in the study by Diatchenko, et al.^38^ (2005), who evaluated pain sensitivity in 202 women using a numerical pain rating scale. Thus, the main result reveals that carriers of the C wild-type allele increase the perception of pain intensity. In this sense, the C allele in rs4818 seems to be a *risk allele* for pain perception.^40^

In this study, self-reported pain was investigated using the FPS-R, which, although not explicitly developed for assessing self-reported pain of dental origin, has been previously administered^41–46^ and is considered valid and reliable. The interpretation of this instrument could be influenced by the cognitive level of the children or the experience of pain from other origins. Thus, to enhance the accuracy of pain experiences of dental origin and circumvent this possible limitation, pain was also measured based on the reports of parents/guardians, which confirmed the veracity of the results obtained using the FPS-R. In fact, the used scale has already proved to be efficient in investigating facial expression and pain perception, since the main strength of the scale is that it reduces false-negative results and can be used in clinical practice.^45^

Finally, genetic studies that use the allelic analysis are essential research tools for mapping a particular gene region and detecting harmful alleles. However, only two alleles are evaluated, which limits the use of such studies. Therefore, combining multiple genetic models, like haplotype analysis, can be an important tool to better understand the role of a gene in the origin and evolution of a disease. In this sense, future studies should perform a haplotype analysis with the inclusion of one or more polymorphisms, which could significantly strengthen the association between the *COMT* gene and dental pain.

## Conclusion

In conclusion, the rs4818 polymorphism in the *COMT* gene was associated with pain of dental origin in the children studied, and individuals with the C allele were more likely to report higher levels of pain.

## Data Availability

All data generated and analyzed during this study are included in this published article.
